# Phenotypic Characterization of CD4^+^ T Lymphocytes in Periportal Fibrosis Secondary to Schistosomiasis

**DOI:** 10.3389/fimmu.2021.605235

**Published:** 2021-02-22

**Authors:** Jordana Batista Santana, Tarcísio Vila Verde Santana de Almeida, Diego Mota Lopes, Brady Page, Sergio Costa Oliveira, Irismá Souza, Luís Eduardo Viana Silva Ribeiro, Néstor Adrián Guerrero Gutiérrez, Edgar M. Carvalho, Luciana Santos Cardoso

**Affiliations:** ^1^ Serviço de Imunologia, Hospital Universitário Professor Edgard Santos, Universidade Federal da Bahia, Salvador, Brazil; ^2^ Massachusetts General Hospital, Boston, MA, United States; ^3^ Departamento de Bioquímica e Imunologia, Instituto de Ciências Biológicas, Universidade Federal de Minas Gerais, Belo Horizonte, Brazil; ^4^ Instituto Nacional de Ciência e Tecnologia em Doenças Tropicais (INCT–DT/CNPq), Salvador, Brazil; ^5^ Instituto de Saúde Irismá Souza, Gandu, Brazil; ^6^ Laboratório de Pesquisas Clínicas, Instituto Gonçalo Moniz, Fundação Oswaldo Cruz (FIOCRUZ), Salvador, Brazil; ^7^ Departamento de Análises Clínicas e Toxicológicas, Faculdade de Farmácia, Universidade Federal da Bahia (UFBA), Salvador, Brazil

**Keywords:** schistosomiasis, periportal fibrosis, *Schistosoma mansoni*, CD4^+^ T lymphocytes, fibrosis

## Abstract

Schistosomiasis is a parasitic disease that affects about 166 million people around the world. It is estimated that 5%–10% of individuals with schistosomiasis develop severe forms of the disease, which are characterized by pulmonary hypertension, ascites, periportal fibrosis, and other significant complications. The chronic phase of the disease is associated with a Th2 type immune response, but evidence also suggests there are roles for Th1 and Th17 in the development of severe disease. The aim of this study was to evaluate the CD4^+^ T lymphocyte profile of patients with different degrees of periportal fibrosis secondary to schistosomiasis. These individuals had been treated for schistosomiasis, but since they live in a *S. mansoni* endemic area, they are at risk of reinfection. They were evaluated in relation to the degree of periportal fibrosis and classified into three groups: without fibrosis or with incipient fibrosis (WF/IFNE), n=12, possible periportal fibrosis/periportal fibrosis, n=13, and advanced periportal fibrosis/advanced periportal fibrosis with portal hypertension, n=4. We observed in the group without fibrosis a balance between the low expression of Th2 cytokines and high expression of T reg cells. As has already been described in the literature, we found an increase of the Th2 cytokines IL-4, IL-5, and IL-13 in the group with periportal fibrosis. In addition, this group showed higher expression of IL-17 and IL-10 but lower IL-10/IL-13 ratio than patients in the WF/IFNE group. Cells from individuals who present any level of fibrosis expressed more TGF-β compared to the WF/IFNE group and a positive correlation with left lobe enlargement and portal vein wall thickness. There was a negative correlation between IL-17 and the thickness of the portal vein wall, but more studies are necessary in order to explore the possible protective role of this cytokine. Despite the fibrosis group having presented a higher expression of pro-fibrotic molecules compared to WF/IFNE patients, it seems there is a regulation through IL-10 and T reg cells that is able to maintain the low morbidity of this group.

## Introduction

Among parasitic diseases in tropical and subtropical regions, schistosomiasis is the second most important in terms of socioeconomic and public health impact. It affects about 240 million people living in developing countries, especially in rural and peri-urban areas, with an estimated 700 million people at risk worldwide (1).

It is estimated that 5%–10% of individuals infected with *S. mansoni* develop severe forms of the disease, which can be characterized by hepatic fibrosis and portal hypertension, ascites, as well as esophageal and gastric varices, which predispose infected individuals to gastrointestinal hemorrhage and death (2, 3). Hepatic fibrosis occurs in response to antigens present on the ova of the parasite, which become lodged in the second-order periportal venous branches, precipitating an inflammatory reaction that leads to granuloma formation with eventual tissue fibrosis (4, 5).

The immunopathogenesis of the schistosomal granuloma is predominantly Th2, characterized by the production of IL-4, IL-5, and IL-13, as reviewed by Wilson et al. (5, 6). There are few studies in the literature showing the involvement of Th1 and Th17 cytokines in the pathogenesis of fibrosis. These studies point to a dual role of IFN-γ cytokine in both pathogenesis and fibrosis protection and focus primarily on experimental models and serum levels of these cytokines *in vitro* (7–9). Little is known about the protective immune response in periportal fibrosis in humans (10). Unlike the results found in a murine model, where IL-10 plays a key role in controlling the inflammatory response in fibrosis (11), *in vitro* studies evaluating the frequency of monocytes from individuals with different degrees of periportal fibrosis do not show an increase in IL-10 production (12). The same result was shown when levels of IL-10 in serum from individuals with different degrees of periportal fibrosis were analyzed (13–15). These results suggest that there are other regulatory sources of the inflammatory process observed in the pathogenesis of fibrosis besides IL-10.

The aim of this study was to characterize the profile of TCD4^+^ lymphocytes expressing Th1, Th2, Th17 cytokines and molecules associated with a regulatory response by individuals with different degrees of periportal fibrosis secondary to schistosomiasis. The identification of a phenotypic profile in peripheral blood lymphocytes of individuals with periportal fibrosis could help in the discovery of immunomodulatory molecules capable of controlling the exacerbation of the fibrosis-associated inflammatory process. The results obtained here open the field for interventions with potential immunomodulatory drugs.

## Methods

### Selection of Participants in the Schistosomiasis-Endemic Region and Study Design

The study was conducted in a small village of Água Preta in the state of Bahia, Brazil. The sanitation conditions of the region are precarious, placing residents at high risk of parasitic infection. The main source of income within the population is agriculture, and river water is used for bathing, washing clothes and utensils, and leisure activities. The population’s access to health services is limited; there are only reports of sporadic previous treatments with anthelmintics.

In this study we included 29 patients with different degrees of periportal fibrosis due to *Schistosoma mansoni* infection, classified using the WHO-Niamey protocol by a trained physician. Interpretation of ultrasonographic results was performed by the combination of numerical results of IP (Image Pattern) scores, PT (Periportal Thickening) scores, and PH (Portal Hypertension).

The examination was carried out by a trained ultrasound specialist. The evaluation was carried out with a portable SONOSITE TITAN device (Medsonic) with convex transductor of 2.0–5.0 Mhz. The interpretation of the results was given by the interpolation of the numerical results of the scores from the liver parenchyma (IP) pattern, in addition to by the mean total thickness of four portal tracts after the first division from the right and left branches of portal vein (PT). The portal hypertension (PH) score was calculated by measuring the internal diameter of the portal vein and the presence of collateral circulation and ascites (1). Study carried out by Santos et al. (16) using the Niamey criteria, demonstrated moderate to substantial intra and inter-observer reproducibility in PPF classification.

Due to small sample sizes, it was also necessary to group together some categories of fibrosis for this stage of the analysis. Patients classified as without fibrosis (WF) and incipient fibrosis not excluded (IFNE) were united in a single group, as were possible periportal fibrosis (PPF) and periportal fibrosis (PF). The groups of advanced periportal fibrosis (APF) and APF with portal hypertension (APF/PH) were also analyzed in the same group. Twelve patients were classified as WF/IFNE, thirteen individuals as PPF/PF, and in the endemic areas we found only four individuals classified as APF/PH.

Individuals of both genders, aged between 10 and 60 years, who already tested positive for *S. mansoni* were included in the immunological evaluation. Subjects less than 10 years of age or older than 60, pregnant women, individuals with a history of chronic alcoholism, individuals with diseases that may interfere with the results of the immune response, such as hepatitis B and C, diabetes mellitus and HIV, and individuals who were on immunosuppressive drugs or undergoing chemotherapy of any kind were excluded from the study. Children younger than 10 and adults over 60 were not included in the immunological evaluation because of the potential of aberrant immune responses in these age groups. The mean age of the APF/PH group was higher (60.7 ± 4.2) than the WF/IFNE group (30.7 ± 10.2; p <0.05). There were no significant differences in gender distribution.

Determination of parasite infection was carried out using the spontaneous sedimentation technique (Hoffmann-Pons-Janer or Lutz method) and determination of the parasite load of *Schistosoma* infection was accomplished by the Kato-Katz method. The majority of individuals reported that they had been treated for schistosomiasis for less than 1 year (98%). This report may justify the low prevalence of infection (15%) at the time of recruitment for ultrasound analysis (USG). Additionally, the prevalence of specific soluble eggs antigen (SEA)-IgE levels was 89% among all groups evaluated (**Table 1**).

**Table 1:** Demographical and clinical characteristics of studied population.

	WF/IFNE (n = 12)	PPF/PF (n = 13)	APF/PH (n = 4)	p value
Age (years)* (median ± SD)	30.7 ± 10.2	35.0 ± 10.9	60.7 ± 4.1	<0.05^b,c^
Female gender n (%)**	6 (50)	8 (61.5)	3 (75.0)	ns
**SEA-Specific IgE (%)**	75%	67%	67%	ns
**Size of left lobe (mm)** **(mean ± SD)**	92.2 ± 12.4	94.0 ± 11.3	119.3 ± 21.1	ns
**Periportal wall measurement (mm)** **(mean ± SD)**	3.5 ± 0.71	4.5 ± 1.2	4.9 ± 1.3	0.0007^a,b^
Treatment report less than 1 year (%)**	91.7	100	75	<0.0001^a,b,c^

*Kruskal-wallis test; ** Fisher’s exact test; WF/IFNE, without fibrosis/incipient fibrosis not excluded; PPF/PF, possible periportal fibrosis/periportal fibrosis; APF/PH, advanced periportal fibrosis/advanced periportal fibrosis + portal hypertension.

^a^WF/IFNE vs. PPF/PF; ^b^WF/IFNE vs. APF/PH; ^c^PPF/PF vs. APF/PH. ns, not significant (p>0.05).

### Ethics Statement

The present study is part of a study approved by the Ethics Committee of the School of Nursing, Federal University of Bahia, entitled: ‘‘Identification of biomarkers associated with the development of severe forms of schistosomiasis’’ (License number: 1,374.864). All individuals who agreed to participate in the study signed the Informed Consent Form.

### Preparation of Peripheral Blood Mononuclear Cells and Flow Cytometry Experiments

Peripheral blood mononuclear cells of patients different degrees of periportal fibrosis due to schistosomiasis were obtained using the Ficoll-Hypaque density gradient technique (GE Healthcare, Uppsala) and adjusted to a concentration of 1 × 10^7^ cells/ml in complete RPMI 1640 (100 μl/ml gentamicin, 2mM L-glutamine, 30mM HEPES) containing 10% heat-inactivated fetal bovine serum (FBS) (Life Technologies GIBCO BRL, Gaithersburg, MD). Cells were stimulated with 10μg of soluble egg antigen (SEA) and maintained at 37°C and 5% CO_2_ in 96-well culture plates for 16h.

After incubation, brefeldin A (10 μg/ml; Sigma, St. Louis, MO) was added for 4 h at 37°C and 5% CO_2_. Labeling of PBMC surface markers was then performed with 20 μl of solution containing the specific conjugated antibodies for CD4 T lymphocytes and activation and regulation markers: CD3 (clone OKT3, eBioscience), CD4 (clone OKT4, eBioscience), CD25 (clone BC96, eBioscience) and CTLA-4 (clone 14D3, eBioscience). After this step, the plates were incubated with 150 μl/well of permeabilization buffer for 10 min at room temperature. After incubation, intracellular labeling was done using anti-cytokine and anti-transcription factor monoclonal antibodies: IL-4 (clone 8D4-8, BD Bioscience), IL-5 (clone TRFK5, eBioscience), IFN-γ (clone 45.B3, eBioscience), IL-17 (clone eBio64DEC17, eBioscience), FOXP3 (clone 236A/E7, eBioscience), IL-13 (clone PVM13-1, eBioscience), IL-10 (JES3-9D7, eBioscience), and TGF-β (clone 9016, R&D System). The acquisition was performed using the FACSCanto (Becton Dickinson) apparatus, for a total of 200,000 events.

**Figure 1:**
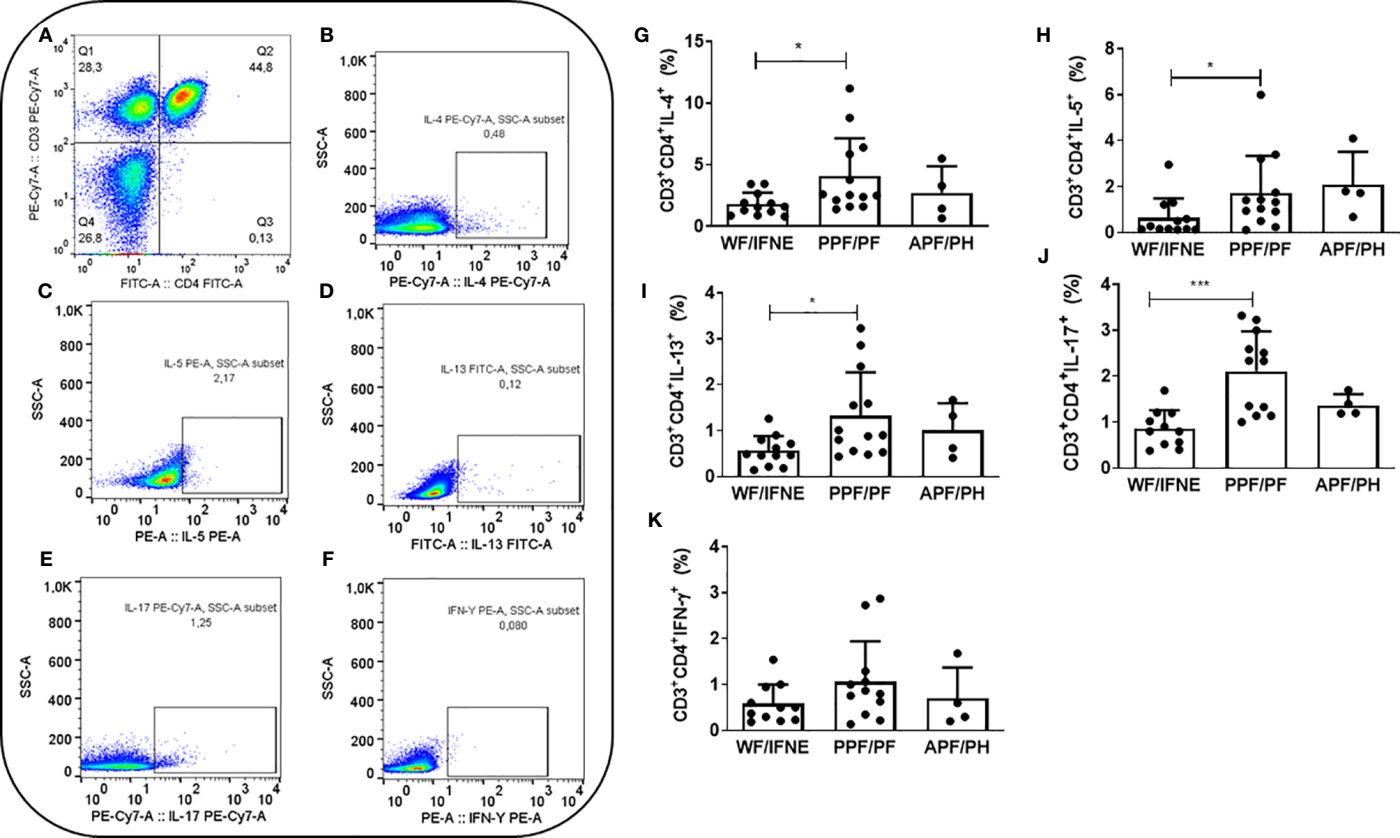
Representative plots of one experiment showing the frequency of CD4^+^ T lymphocytes **(A)** and the expression of IL-4 **(B)**, IL-5 **(C)**, IL-13 **(D)**, IL-17 **(E)**, and IFN-γ **(F)**. Frequency of CD4^+^ T lymphocytes expressing IL-4 **(G)**, IL-5 **(H)**, IL-13 **(I)**, IL-17 **(J)**, IFN-γ **(K)** after SEA stimulation from individuals without fibrosis and with different degrees of periportal fibrosis secondary to schistosomiasis *p <0.05, ***p <0.005 (Mann-Whitney test).The histogram and bars represent the mean + SD.

The Flow Jo V10 (Tree Star, BD) program was used for analysis of lymphocytes. These cells were analyzed according to the frequency of expression of cell surface markers. Cell populations were defined by non-specific fluorescence from frontal (FSC) and lateral light (SSC) dispersion by parameters of particle size/volume and particle complexity, respectively. A population of lymphocytes was selected by gating based on cellular characteristics in this population. A specific region was delineated in the graph corresponding to the lymphocyte area, then the T lymphocyte population was selected by the presence of CD3 (**Figure 1A**), and within this population the CD4^+^ T lymphocyte population was subsequently identified. We finally evaluated the expression surface markers and intracellular cytokines in this population (**Figures 1B–F**).

### Schistosoma mansoni Soluble Egg Antigen

The *S. mansoni* soluble egg antigen (SEA) used in this study were prepared as previously described (17) and kindly provided by Dr. Alfredo M. Góes.

### Determination of Cytokine Levels

Levels of IL-10 and IL-13 were evaluated in the supernatants of PBMC cultures stimulated with SEA antigen (10 µg/ml) for 16 h according to the manufacturer’s instructions (Pharmingen, San Diego, CA). Briefly, plates (Nunc-Immuno Plate MaxiSorp Surface, Denmark) were sensitized overnight at 4°C with human anti-cytokine monoclonal antibody (anti-IL-10 or anti-IL-13). The following day, after washing the plates with PBS/Tween 0.05%, blockade of non-specific binding was performed with PBS + 0.01% bovine albumin for 2 h at room temperature. Three washes were performed with PBS/Tween 0.05% and then the samples, blanks, and standards were added and the plate was incubated at room temperature for 2 h. The plate was washed again 3 times and biotinylated human anti-cytokine detection antibody (2 μg/ml) was added. After incubating for 1 h at room temperature the plates were washed 4 times and the conjugate (streptavidin-conjugated peroxidase) was added. The plate was incubated for 30 min at room temperature. After washing, the substrate (3,3’,5,5’-tetramethylbenzidine + H2O2 + dimethyl sulfoxide) was added and the plate was incubated for 20 min at room temperature. The reaction was interrupted by the addition of H2SO4 (8M). Optical density (OD) was read at 450 nm (SpectraMax, Molecular Devices Corporation, Sunnyvale, CA) and the values were converted to pg/ml based on the standard curve (Soft Max Pro 5.0 Molecular Devices Corporation, Sunnyvale, CA).

### Sample Size and Statistical Analysis

Sample size was calculated based on previous studies in which the degree of periportal fibrosis was evaluated by the Cairo protocol. We observed that 25% of individuals presented some degree of periportal fibrosis (18). A study power of 80% and an α error of 0.05% were taken into account. One blood sample was collected from each individual. The samples were collected in different trips to the endemic area and the data were plot in the same graph. Statistical analysis was performed in GraphPad PRISM 5.0 (La Jolla, CA, USA). Before the analysis of each data set, the D’Agostino-Pearson normality test was performed. Therefore, for comparison between two or more groups, parametric and non-parametric tests were used according to the nature of the data generated (ANOVA, Kruskal Wallis). Spearman correlation (r_s_) was calculated in the correlation analysis. All tests were two-tailed and statistical significance was established in the 95% confidence interval. P values <0.05 were considered significant.

## Results

### Cytokine Profile Expressed by CD4^+^ T Lymphocytes From Patients With Different Degrees of Periportal Fibrosis Secondary to Schistosomiasis

To investigate the contribution of Th1, Th2, and Th17 cytokines, we analyzed the intracellular expression of these molecules in CD4^+^ T lymphocytes from individuals with periportal fibrosis secondary to schistosomiasis. We first evaluated the frequency of T CD4^+^ lymphocytes expressing IL-4, IL-5, and IL-13 after stimulation with SEA, since these cytokines are the main molecules involved in the pathogenesis of periportal fibrosis related to *Schistosoma mansoni* infection (19). We observed an increase in the frequency of CD4^+^ T cells expressing these cytokines in the group of individuals with periportal fibrosis (PPF/PF), compared to individuals without fibrosis (WF/IFNE) (**Figures 1G–I**).

There was an increase in the frequency of CD4^+^ T lymphocytes expressing IL-17 in the PPF/PF group (p<0.01), when compared to WF/IFNE group (**Figure 1J**). In order to try to understand the possible role of this cytokine we proceeded to correlate its expression with ultrasonographic specific measurements. We performed a correlation between IL-17 and the measurement of the periportal wall and the size of the left lobe (**Supplementary Figure 1A**) and observed a negative correlation between the frequency of TCD4^+^IL-17^+^ cells and the measurement of the periportal wall (r_s_ =0.53; p <0.05), but we did not observe any correlation with the size of the left lobe (**Supplementary Figure 1B**). The intracellular expression of IFN-γ in CD4 T lymphocytes did not differ between all evaluated groups (**Figure 1K**).

### Evaluation of Regulatory Molecules by CD4 T Lymphocytes in Individuals Without Fibrosis and With Different Degrees of Periportal Fibrosis

**Figure 2:**
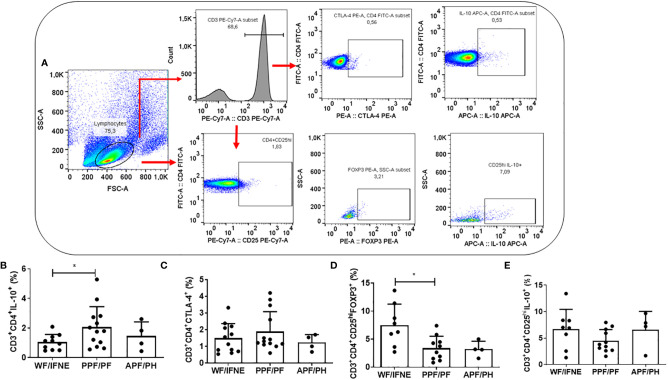
Flow cytometry gating strategy used in order to analyze the regulatory molecules, CTLA-4, FOXP3 and IL-10 **(A)**. Frequency of CD4 T lymphocytes expressing IL-10 **(B)**, CTLA-4 **(C)**, and the CD4+CD25+ that express FOXP3 **(D)** or IL-10 **(E)** after SEA stimulation from individuals without fibrosis and with different degrees of periportal fibrosis secondary to schistosomiasis *p < 0.05, (Mann-Whitney test). The histogram and bars represent the mean + SD.

We next evaluated the frequency of the main regulatory molecules involved in the control of schistosomiasis fibrosis (6) CTLA-4, IL-10, besides regulatory T cells (CD4^+^CD25^hi^) expressing the regulatory molecules FOXP3 and IL-10 (**Figure 2A**).

The frequency of CD4^+^IL-10^+^ T lymphocytes was higher in the PPF/PF group, compared to the WF/FINE group (p<0.01; **Figure 2B**). We did not observe any difference in the CD4^+^CTLA-4^+^ T cells among all groups (**Figure 2C**). The frequency of T reg FOXP3^+^ cells was higher in the group of individuals WF/FINE compared to PPF/PF (p<0.01; **Figure 2D**). Regarding the expression of IL-10 by the regulatory cells, in our study we did not find any statistical difference among all groups (**Figure 2E**).

Since we observed in the group without fibrosis a lower frequency of TCD4^+^ lymphocytes expressing IL-10, due to its role in controlling the pathogenesis of the disease, we evaluated the levels of this cytokine in the culture supernatant (**Supplementary Figure 2A**. The levels of IL-10 did not differ between the WF and PPF/PF groups, while they were lower in the APF/PH group, compared to the PPF/PF group (p<0,01; **Supplementary Figure 2A**).

We performed a correlation between CD4^+^ T cells expressing IL-10 with CD4^+^ T lymphocytes expressing IL-4 or IL-13. We observed a positive correlation only with CD4^+^IL-4^+^ T cells (p=0.0007, r=0.79) (**Supplementary Figure 3A**).

We then evaluated the levels of IL-13 in the culture supernatants (**Supplementary Figure 2B**), one of the main cytokines related to the fibrosis process (13, 19, 20); the results were similar to the expression of IL-13 by CD4^+^ T lymphocytes (**Figure 1I**). However, the IL-10/IL-13 ratio was higher in the group without fibrosis, compared to the PPF/PF group (p <0.01; **Supplementary Figure 2C**). These findings suggest that IL-10 produced by other cellular sources, such as macrophages, dendritic cells and CD8^+^ T lymphocytes, would be of fundamental importance in the regulation of this group without fibrosis.

### The Expression of Pro-Fibrotic TGF-β by CD4^+^ T Lymphocytes and Correlation With the Pathogenesis of Periportal Fibrosis Secondary to Schistosomiasis

**Figure 3:**
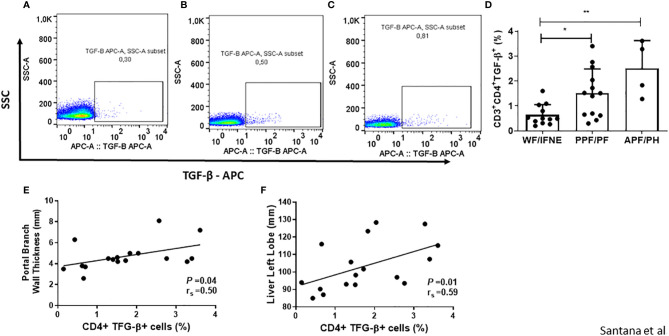
Representative plots of one experiment showing the frequency of CD4^+^TGF-β^+^ T lymphocytes in the different groups **(A–C)**. Frequency of CD4 T lymphocytes expressing TGF-β after SEA stimulation from individuals without fibrosis and with different degrees of periportal fibrosis secondary to schistosomiasis **(D)**. Correlations between this value and the portal branch wall thickness or the size of the liver left lobe of the patients that shows some level of fibrosis **(E, F)**. Spearman’s rank correlation coefficient (r_s_) and *P* value is shown. *p < 0.05 **p < 0.01.

Since TGF-β is the main pro-fibrotic cytokine produced by CD4^+^ T lymphocytes, we decided to evaluate the frequency of these cells producing TGF-β (**Figures 3A–D**) and the correlation between this cytokine and ultrasonography measurements (**Figures 3E, F**).

We observed a higher frequency of CD4^+^TGF-β^+^ T lymphocytes in the cultures of individuals with any grade of periportal fibrosis, both in the PPF/PF and APF/PH groups, compared to the group without fibrosis (p<0.01; **Figure 3A**). We also performed a correlation between TCD4^+^ TGF-β^+^ T lymphocytes from cultures of individuals with some degree of periportal fibrosis with the measurement of the periportal wall and the size of the left lobe (**Figures 3E, F**). And we observed a positive correlation both with the measurement of the periportal wall (r=0.5; p<0.05) and the size of the left lobe (r=0.59; p<0.05). We did not observe a significant difference in correlation of the frequency of CD4^+^T cells expressing TGF-β with CD4^+^ IL-4^+^ and CD4^+^IL-13^+^ T cells in the group of individuals with periportal fibrosis (**Supplementary Figure 3B**).

## Discussion

The hepatic fibrosis associated with chronic *S. mansoni* infection contributes to the development of the most severe forms of the disease and is responsible for the morbidity and mortality observed in individuals living in endemic areas (6). In this study, we identified the complex network of cytokines expressed by CD4^+^T lymphocytes in individuals with periportal fibrosis secondary to schistosomiasis who reside in an endemic area.

Epidemiological studies in populations living in endemic areas suggest that the development of hepatic fibrosis is associated with several factors such as disease duration, parasite load, sex, as well as genetic and immunological factors of the study population (4, 21). Demographic analysis of the study population showed no difference in gender ratio between individuals evaluated by ultrasonography and those selected for immunologic evaluation. Some studies, however, have demonstrated the importance of gender in the development of periportal fibrosis (22, 23). In fact, a previous study by our group in the Água Preta population showed that women were more likely than men to be exposed to contaminated water, probably due to gendered activities taking place in river water such as washing clothes, dishes, and food preparation. Despite this, no significant differences in the development of hepatic fibrosis between genders in this population were found (10).

As far as age, the APF/PH group presented a higher median age in relation to the WF/IFNE group. These results corroborate the data presented by Alves Oliveira et al. (15) in which the majority of individuals with the most advanced degrees of fibrosis were over 50 years of age (15). This could be explained by repeated parasite re-exposure in the older age group or by the slow process of fibrotic tissue generation; younger individuals have likely not been exposed enough time for the cumulative effects of the excessive secretion of collagen in the peripheral hepatic parenchyma or near the periportal tract to be observed (24).

The present study demonstrates a greater production of IL-4, IL-5, and IL-13 by the lymphocytes of individuals with periportal fibrosis. IL-4 is responsible for the differentiation of the Th2 response and the blockade of this cytokine in *S. mansoni*-infected mice leads to a decrease in hepatic collagen deposition (25). In animals reinfected by *S. mansoni*, IL-4 is a risk factor for the development of fibrosis and in humans this cytokine shows a positive correlation with fibrosis score (26, 27). There are studies that also strengthen the association between a higher degree of fibrosis and higher levels of IL-5, a cytokine whose main function is eosinophil activation (13). Additionally, IL-5 knockout is able to reduce hepatic fibrosis in *S. mansoni*-infected mice (28). Many studies have pointed to IL-13 as the main pro-fibrotic cytokine, since its blockade is capable of both preventing the onset of periportal fibrosis and of reducing already established fibrosis (17, 29, 30). IL-13 levels in the supernatant of PBMCs cultures from patients also show a positive association with the presence and severity of periportal fibrosis (15). In this study, the group with fibrosis showed a higher frequency of CD4^+^ T lymphocytes expressing IL-13 when compared to individuals without fibrosis, as well as higher levels of this cytokine in the supernatant of PBMCs cultures stimulated with SEA. The source of Th2-type cytokines are not only produced by Th2 cells, but are also secreted by other innate lymphocytes, such as type 2 innate lymphoid cells (ILC2s), which could be contributing to the higher levels of this cytokine in the culture supernatant.

Contrary to what was expected, lymphocytes from individuals with advanced fibrosis did not present a higher production of Th2 and proinflammatory cytokines when compared to the other groups. This fact may be associated with the phenomenon of immune exhaustion, which is characterized by the gradual and progressive loss of specific T-cell effector functions due to antigenic persistence in chronic infections (31). Despite being initially described in models of viral infections, exhaustion has also been documented in parasitic infections, including schistosomiasis in a murine model, as reviewed by Rodrigues et al. (32). Another fact corroborating this hypothesis is the advanced age of patients with advanced fibrosis, which may indicate a longer period of infection and antigenic exposure, that would lead to hyposensitivity to the parasite. However, we must consider the small number of individuals with the most severe forms of fibrosis in the endemic area.

Regarding the expression of IL-17 by CD4^+^ T lymphocytes; interestingly, lymphocytes of individuals with fibrosis had a higher production of IL-17 (6). However, the role of IL-17 in periportal fibrosis secondary to schistosomiasis is still poorly understood. IL-17 is able to exacerbate fibrosis in the murine model through activation of Kupffer cells and direct induction of collagen production by hepatic stellate cells (33). Neutralization of IL-17 is able to reduce granuloma formation and liver damage in mice infected with *S. japonicum* (34). This evidence, when viewed in conjunction with our data, leads us to believe that IL-17 acts as a pro-fibrotic cytokine in schistosomiasis, as could be the case with *S. haematobium* (35). In addition to the expression of IL-17A by CD4^+^ T lymphocytes, we expanded the focus of our study by assessing the correlation between this cytokine and parameters obtained by ultrasonography. There is a negative correlation between CD4^+^T IL-17^+^ and the thickness of the portal vein wall, suggesting a protective role of this cytokine in fibrogenesis. This could be due to the fact that Th17 is not the only source of the cytokine (36). Further studies are needed to clarify the role of this cytokine in the protection or pathogenesis of periportal fibrosis in humans.

The expression of the proinflammatory cytokine IFN-γ by lymphocytes of individuals did not differ among all groups. The role of this cytokine in periportal fibrosis secondary to schistosomiasis remains controversial, as the majority of studies show a profibrotic role for this cytokine, as reviewed by Zheng et al. (6).

The cytokine IL-10 seems to have an important protective role in the pathogenesis of periportal fibrosis. We observed a higher frequency of CD4^+^IL-10^+^ T cells in the PPF/PF group compared to individuals without fibrosis. The increased expression of these cellular markers in the PPF/PF groups could be explained by an attempt to control the greater activation and production of Th2 and proinflammatory cytokines observed in the lymphocytes of this group of patients. The smaller ratio of secreted cytokines IL-10/IL-13 could indicate that the regulatory response is not strong enough to prevent the fibrosis. Different studies have shown that the low level of IL-10 secreted by PBMCs was associated with severe fibrosis (24, 37). And there could be additional sources of IL-10-like monocytes. We have found a correlation between the frequency of CD4^+^ T cells expressing IL-4^+^ with CD4^+^ T cells expressing IL-10^+^ or TGF-β. A study evaluating PBMC from patients with filariasis, showed that CD4^+^ T cells are the main source of IL-10 and the majority of these cells co-produced neither IL-4 nor IFN-γ. In this study, only 22% of them stained positively for IL-4 (38). In our study, the persistence of the SEA antigen in the second periportal branches could justify the positive correlation observed between IL-4 and IL-10. And IL-10 could also be produced by Th2 cells and other cells, like macrophages. Further investigations, analyzing in detail which specific cell populations are expressing each cytokine, using a flow cytometry approach that could include these types of analysis, may help us to understand the role of these populations.

Few studies have focused on the profile of T reg cells in individuals with periportal fibrosis secondary to schistosomiasis, and how they become activated and migrate to exert their functions has been reviewed elsewhere (39). In our study, we observed a higher frequency of T reg lymphocytes in the group without fibrosis compared to the PPF/PF group. In a previous work we have shown a higher frequency of CD4^+^CD25^hi^ T cells in individuals with moderate/severe fibrosis (10). Other studies have associated the presence of T reg FOXP3^+^ lymphocytes in peripheral blood with the worsening of the disease, suggesting a failure in the recruitment of this cell population to the liver tissue (40, 41).

Regarding TGF-β, an important cytokine involved in fibrosis due to its ability to induce fibroblast proliferation and collagen deposition, we observed a higher frequency of CD4^+^ T lymphocytes expressing this cytokine in groups with some degree of fibrosis. Studies evaluating PBMCs stimulated with SEA or serum levels of TGF-β from individuals with different degrees of fibrosis did not report differences between the studied groups (13, 14). Nonetheless, in a study published by our group in 2014 evaluating subpopulations of monocytes in patients with different degrees of fibrosis secondary to schistosomiasis, we observed higher intracellular levels of TGF-β in classic, intermediate, and non-classic monocytes in individuals with moderate to severe fibrosis compared to individuals without fibrosis or individuals with incipient fibrosis (12). Our findings suggest that like monocytes, the CD4^+^ T lymphocytes are an important source of TGF-β and play an essential role in fibrogenesis. Our results also show a positive correlation between TGF-β and the measurement of the left lobe and the portal vein wall thickness. The article published by Li et al. (42) analyzing individuals with periportal fibrosis due to *Schistosoma japonicum* infections shows a positive correlation between TGF-β mRNA levels with spleen thickness and liver stiffness (42), parameters which are correlated with the severity of the disease. Furthermore, an association between the expression of this molecule and the degree of inflammation was observed; therefore, TGF-β could participate in both the inflammatory process and the fibrotic process (42–44).

In light of the above, we conclude that in the PPF/PF group, despite intense production of cytokines associated with the inflammatory response, there is a regulation through IL-10 and T reg cells that could maintain the low morbidity of this group. To our knowledge, this is the first study showing the complex network of cytokines expressed by CD4^+^ T cells in endemic area patients classified by ultrasonography. Interventions focused on the control of the cytokines related to periportal fibrosis could be useful to reduce the morbidity associated with schistosomiasis.

## Data Availability Statement

The raw data supporting the conclusions of this article will be made available by the authors, without undue reservation.

## Ethics Statement

The studies involving human participants were reviewed and approved by the Ethics Committee of the School of Nursing, Federal University of Bahia, License number: 1,374.864. The patients/participants provided their written informed consent to participate in this study.

## Author Contributions

LC, EC, and SO contributed to conception and design of the study. JS, TA, DL, LR and BP carried out most of the experiments. IS performed the ultrasound evaluations of the patients and helped in the interpretation of the results. JS, BP, NG, and LC wrote the manuscript and carried out the statistical analysis and prepared the figures. LC submitted this paper. All authors contributed to the article and approved the submitted version.

## Funding

This work was supported by the FAPESB/EDITAL UNIVERSAL, grant number APP0051/2016. This work was also supported by the CNPq/MST/INCT-DT, grant number 465229/2014-0. This study was financed in part by the Coordenação de Aperfeiçoamento de Pessoal de Nível Superior - Brasil (CAPES)—Finance Code 001.

## Conflict of Interest

The authors declare that the research was conducted in the absence of any commercial or financial relationships that could be construed as a potential conflict of interest.

## Supplementary Material

Supplementary Figure 1 Correlations between the frequency of CD4^+^CD17^+^ T lymphocytes and the portal branch wall thickness or the size of the liver left lobe of the patients that show some level of fibrosis. Spearman’s rank correlation coefficient (r_s_) and *P* value is shown.

Supplementary Figure 2 Levels of IL-10 **(A)** and IL-13 **(B)** in the supernatant of PBMC stimulated with SEA antigen from individuals without fibrosis and with different degrees of periportal fibrosis secondary to schistosomiasis. **(C)** The IL-10/IL-13 ratio. *p <0.05, (Mann-Whitney test). The bars represent the mean + SD. WF/IFNE, without fibrosis and incipient fibrosis not excluded (N=12); PF/PPF, periportal fibrosis and possible periportal fibrosis (N=12). APF/PH, advanced periportal fibrosis and APF with portal hypertension (N=4).

Supplementary Figure 3 Correlations between the frequency of CD4^+^ T cells from individuals with any degree of periportal fibrosis expressing different cytokines. **(A)** CD4^+^IL4^+^ T cells vs. CD4^+^ IL-10 ^+^; **(B)** CD4^+^IL13^+^ vs. CD4^+^ IL-10^+^; **(C)** CD4^+^IL4^+^ T cells vs. CD4^+^TGF-β ^+^ T cells; **(D)** CD4^+^IL-13^+^ T cells vs. CD4^+^TGF-β ^+^ T cells. Spearman’s rank correlation coefficient (r_s_) and *P* value is shown.
