# Supplementary figures and images for: Phenotypic Characterization of CD4+ T Lymphocytes in Periportal Fibrosis Secondary to Schistosomiasis
Source: Front Immunol. 2021 Feb 22;12:605235. doi: 10.3389/fimmu.2021.605235 (PMC7937650; doi:10.3389/fimmu.2021.605235)

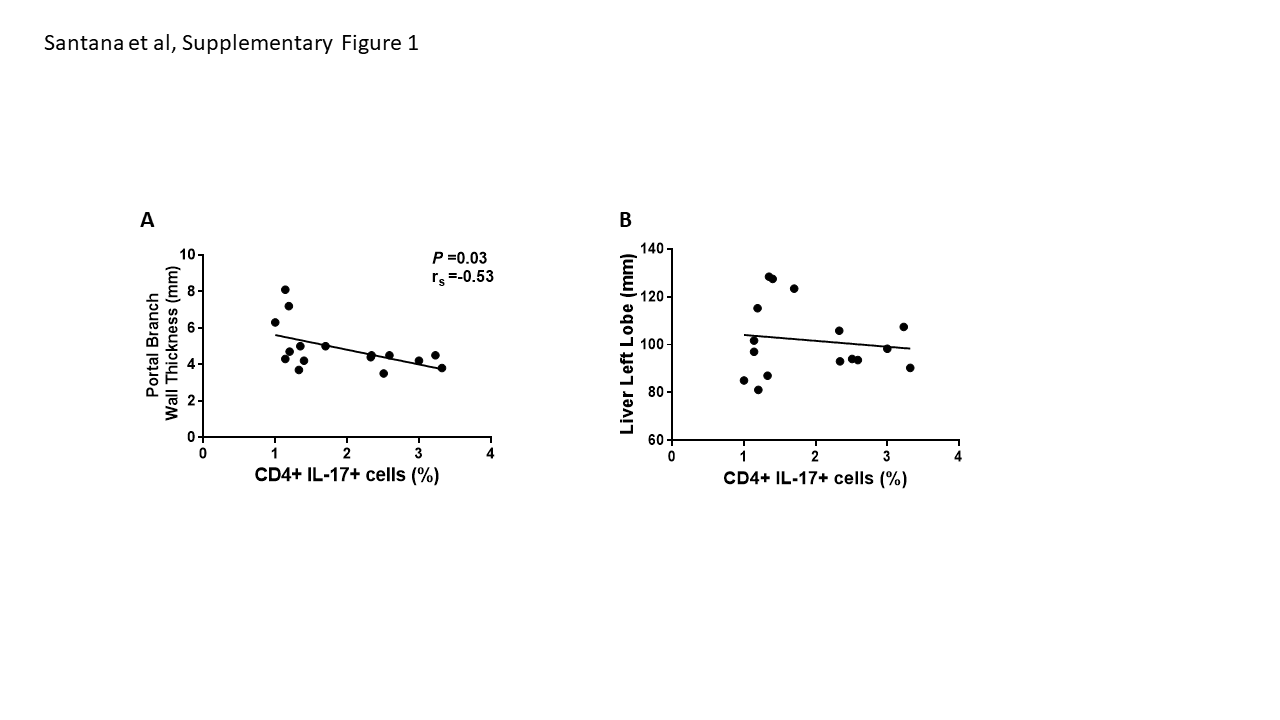

Supplement: Supplementary Figure 1 — Correlations between the frequency of CD4+CD17+ T lymphocytes and the portal branch wall thickness or the size of the liver left lobe of the patients that show some level of fibrosis. Spearman’s rank correlation coefficient (rs) and P value is shown. [file Image_1.tif]

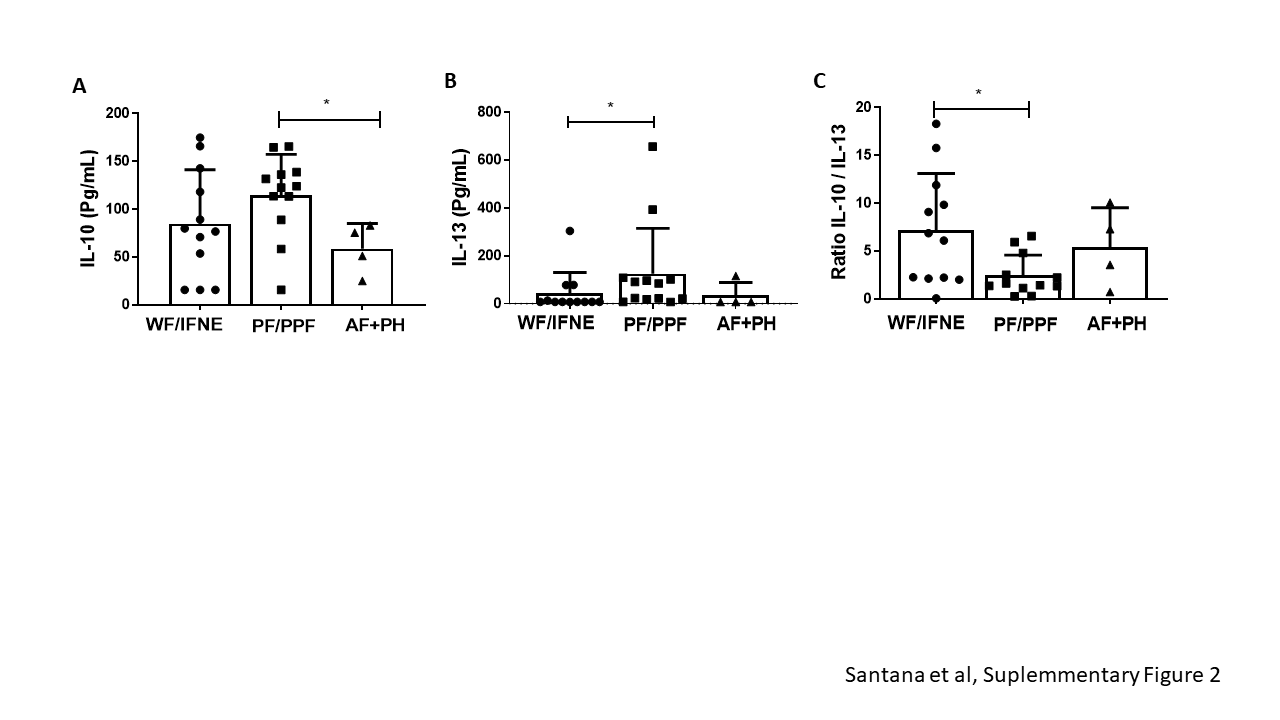

Supplement: Supplementary Figure 2 — Levels of IL-10 (A) and IL-13 (B) in the supernatant of PBMC stimulated with SEA antigen from individuals without fibrosis and with different degrees of periportal fibrosis secondary to schistosomiasis. (C) The IL-10/IL-13 ratio. *p <0.05, (Mann-Whitney test). The bars represent the mean + SD. WF/IFNE, without fibrosis and incipient fibrosis not excluded (N=12); PF/PPF, periportal fibrosis and possible periportal fibrosis (N=12). APF/PH, advanced periportal fibrosis and APF with portal hypertension (N=4). [file Image_2.tif]

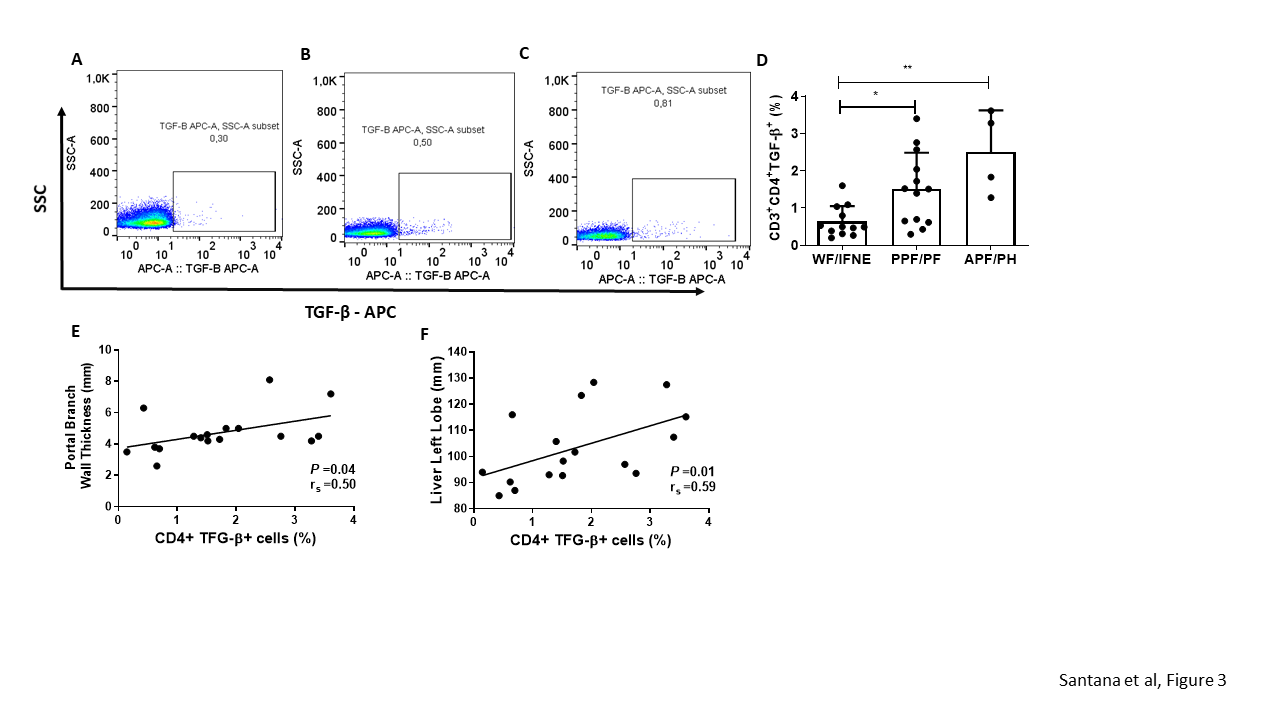

Supplement: Supplementary Figure 3 — Correlations between the frequency of CD4+ T cells from individuals with any degree of periportal fibrosis expressing different cytokines. (A) CD4+IL4+ T cells vs. CD4+ IL-10 +; (B) CD4+IL13+ vs. CD4+ IL-10+; (C) CD4+IL4+ T cells vs. CD4+TGF-β + T cells; (D) CD4+IL-13+ T cells vs. CD4+TGF-β + T cells. Spearman’s rank correlation coefficient (rs) and P value is shown. [file Image_3.tif]
